# Dynamic 18FDG PET/CT and dynamic contrast enhanced MRI of locally advanced breast cancer

**DOI:** 10.1186/1470-7330-14-S1-P14

**Published:** 2014-10-09

**Authors:** O Kupik, M Tuncel, F Demirkazik, M Akpinar, K Altundag, B Erbas

**Affiliations:** 1 Department of Nuclear Medicine, Hacettepe University Medical School, Radiology, Clinical Oncology, Ankara, Turkey

## 

DCE-MRI provides information about the perfusion of tumours together with morphological details. Perfusion of tumour could be assessed by dynamic 18FDG PET/CT (dFDG/PET), in addition to metabolic information. This study was planned to compare the semi-quantitative parameters of DCE-MRI) and dFDG/PET in locally advanced breast cancer.

Forty patients with LABC underwent DCE-MRI and DFDG/PET study at baseline and after 2-3 cycles of neoadjuvant chemotherapy. Tumour longest diameter, spherical (SV), and angiographic volumes (AV) were recorded. Peak signal intensity (PSI), rapid and medium component of initial rise, percentage of Type I, Type II, Type III curves were calculated. Dynamic 18FDG images for the first 30 minutes and late images at second hour were recorded in the prone position, with hanging breasts and the arms above, using a special cushion. Using 18FDG dynamic data, slopes of time-activity curves for the first 2, 5 and 30 minutes were calculated. SUV max and values for 2nd, 5th and 30th minutes were measured. Metabolic tumour volume (MTV) and total glycolytic index (TLG) were calculated for primary lesion and axillary lymph nodes.

Baseline angiographic volume (AV) of DCE-MRIs and metabolic (MTV) volume of 18FDG PET/CT studies were significantly correlated. PSI had a negative correlation with 2nd minute slope of FDG dynamic curve, whereas type III enhancement percentage had positive correlation with 2/30 slope ratio. % AV change showed significant relationship with % SUVmax-bw, % SUVpeak, % MTV and % TLG.

Dynamic imaging with 18FDG provides useful information about tumour perfusion which is in relation with dynamic CE-MRI parameters.

